# Robust Phenotypic Activation of Eosinophils during Experimental *Toxocara canis* Infection

**DOI:** 10.3389/fimmu.2018.00064

**Published:** 2018-01-31

**Authors:** Joice Margareth de Almeida Rodolpho, Luciana Camillo, Márcio Sobreira Silva Araújo, Elaine Speziali, Jordana Grazziela Coelho-dos-Reis, Ricardo de Oliveira Correia, Débora Meira Neris, Olindo Assis Martins-Filho, Andréa Teixeira-Carvalho, Fernanda de Freitas Anibal

**Affiliations:** ^1^Laboratory of Inflammation and Infectious Diseases, Department of Morphology and Pathology, Federal University of São Carlos (UFSCar), São Carlos, Brazil; ^2^Integrated Research Group in Biomarkers, René Rachou Institute (FIOCRUZ), Belo Horizonte, Brazil

**Keywords:** eosinophil, *Toxocara canis*, SLMV, activation, co-stimulatory molecules

## Abstract

Eosinophils are multifunctional cells that have cytotoxic proinflammatory activities and stimulate CD4^+^ T-cells in experimental models of allergy and parasitic infections. Eosinophils, when exposed to antigens, are activated, expressing the CD38/CD69 molecules and exhibited increased expression of major histocompatibility complex (MHC-II), CD80 and CD86, suggesting they play a role upon *Toxocara canis* antigen stimulation. In the present study, we evaluated the profile of eosinophils using conventional and image flow cytometry upon experimental *T. canis* infection. *T. canis* antigens induced a robust activation on this subset, contributing to the immune responses elicited in the experimental model for *T. canis-*associated visceral larva migrans syndrome. Data analysis demonstrated that, during murine *T. canis* infection, eosinophils from peripheral blood, spleen, and bone marrow presented upregulated expression of CD69/MHC-II/CD80/CD86. As opposed to splenic and bone marrow eosinophils, circulating eosinophils had increased expression of activation markers upon *T. canis* infection. The enhanced connectivity between eosinophils and T-cells in *T. canis*-infected mice in all three compartments (peripheral blood, spleen, and bone marrow) also supports the hypothesis that eosinophils may adopt a role during *T. canis* infection. Moreover, *in vitro T. canis* antigen stimulation resulted in activation and upregulation of co-stimulatory-related molecules by bone marrow-derived eosinophils. Our findings are evidence of activation and upregulation of important activation and co-stimulatory-related molecules in eosinophils and suggest a reshape of activation hierarchy toward eosinophils during experimental *T. canis* infection.

## Introduction

Eosinophils are multifunctional cells that have both cytotoxic and proinflammatory activities. When activated, these cells migrate to lymph nodes and activate CD4^+^ T-cells at paracortical zones (1, 2). Upon antigen exposure, eosinophils are apparently activated and express increased levels of CD80, CD86, and major histocompatibility complex (MHC-II) molecules (3–6). In addition, antigen-loaded eosinophils may activate T-cells and increase the production of Th2-type cytokines in several experimental models (7, 8).

Regarding helminthic infections, eosinophils have a protective role, acting as defenders against parasites. In the initial phase of infection, eosinophils are able to capture helminthic antigens and migrate to T cell-rich regions, and present antigen to trigger specific responses (9, 10). In this phase, there is increased influx of newly differentiated eosinophils from the bone marrow to peripheral blood and tissues. In steady-state, bone marrow hematopoietic cells give rise to eosinophil precursor cells and their differentiation and proliferation are regulated by granulocyte-macrophage colony-stimulating factor (GM-CSF) and interleukins such as IL-3 and IL-5 (11).

In regard to nematodes, a role for eosinophils in the pathophysiology of *Toxocara canis* infection has been reported. *T. canis* is the causative agent of canine toxocariasis and the etiological agent of visceral migrans larva syndrome (VLMS) in humans. During *T. canis* infection, eosinophils increase in blood and tissue, which is usually associated with high levels of serum IgE and Th2 immune responses (12–17).

The clinical and pathological aspects of VLMS are multifactorial and seem to be induced by the tissue damage caused by larvae migration. In addition, parasite-derived metabolites and the host inflammatory responses act in consonance to promote granuloma formation in several tissues, mainly in the lung and the liver (14).

Considering the role of eosinophils during helminth infection, these cells could be efficient in activation of specific T-cell responses. During the helminthic infections, eosinophils are able to orchestrate and induce a robust CD4^+^ T-cell activation that is able to control parasite growth. The microenvironment during these infections favor the differentiation of Th2 CD4^+^ T-cell responses, which direct the immune response to these antigens by secretion of inflammatory mediators such as leukotrienes and the cytokines IL-4, IL-5, and IL-13 (18–20).

In this context, the role of eosinophils in *T. canis* infection is poorly understood. Therefore, this study aimed at investigating the *ex vivo* and *in vitro* eosinophil phenotype and function upon *T. canis* exposure. In order to accomplish this goal, we evaluated the changes in the activation status and expression of activation and co-stimulatory surface markers by eosinophils and APC (monocyte/macrophages and B-cells) triggered by *T. canis* experimental infection. In addition, *in vitro T. canis* antigen stimuli were employed to characterize the phenotypic changes in bone marrow-derived eosinophils. Our findings are evidence of activation and upregulation of important activation-related molecules in eosinophils and suggest a reshape of activation hierarchy toward eosinophils during experimental *T. canis* infection.

## Materials and Methods

### Experimental Animals and Parasites

BALB/c mice were purchased from the Faculdade de Ciências Farmacêuticas de Ribeirão Preto at Universidade de São Paulo. All mice were housed in filter top microisolator boxes under light and temperature-controlled conditions. *T. canis* L3 strain was donated by Professor Fabio Ribeiro Braga from the Laboratory of the Experimental Parasitology at the Universidade de Vila Velha (Espírito Santo State, Brazil). Animals were infected with 1,000 eggs/0.3 mL saline by oral gavage as previously described (21). This study was approved by the Ethics Committee on Animal Experimentation at the Federal University of São Carlos under the Protocol number 058/2013. The experimental design is illustrated in Figure S1 in Supplementary Material.

### *Ex Vivo* Phenotypic Evaluation of Eosinophils and T-Cells in Experimental *T. canis* Infection in Mice

On the 18th day post *T. canis* infection, peripheral blood samples were collected in tubes with EDTA by retro-orbital vein puncture. Plasma samples were prepared after centrifugation of blood (15 min at 400 × *g*, 18°C) and stored for further analysis by ELISA. An aliquot of whole-blood cells was utilized for the *ex vivo* flow cytometric analyses of eosinophils as described for spleen and bone marrow cells below.

After peripheral blood collection, *T. canis*-infected and non-infected mice were euthanized and spleens were harvested for preparation of splenocyte suspension. Splenocyte suspension was obtained after maceration using cell strainer and transferred to 15 mL conic tubes. RPMI was added to the tubes and splenocytes were incubated on ice for 15–20 min, centrifuged (7 min. at 400 × *g*, 18°C) and the supernatant was discarded. For preparation of bone marrow cells, femurs and tibias were harvested from *T. canis*-infected and uninfected mice. The femurs and tibia were flushed with PBS to remove the bone marrow from the bone. The flushed bone marrow was gently passed through a cell strainer and washed once. Red blood cells from peripheral blood, spleen, and bone marrow cell suspensions were lysed using RBC lysis buffer (PBS 10×) under soft agitation in vortex. After lysis, 1 mL of PBS 1× was added and cells were washed twice. Cellular viability and concentration were assessed by trypan blue, prior to staining with monoclonal antibodies for cell surface, and activation markers by flow cytometry.

### *In Vitro T. canis* Stimulation of Bone Marrow-Derived Eosinophils

Antigen preparation for stimulation assays were performed using *T. canis* eggs. Briefly, *T. canis* eggs were washed with sterile distilled water, centrifuged (978 × *g*, 5 min at RT) resuspended in 5% sodium hypochlorite and kept for a maximum of 5 min. Eggs were washed with saline three to four times under the same conditions described for complete removal of hypochlorite. After centrifugation (978 × *g*, 5 min at RT), eggs were resuspended in supplemented RPMI medium (10% SBF, 3% streptomycin/ampicillin) and incubated at RT to extract excretory/secretory larvae antigens. The culture supernatant was collected weekly, centrifuged at 2,000 × *g* for 5 min and frozen at −20°C until the moment of use. After removal of the RPMI medium (50 mL) (humidified atmosphere 5% CO_2_, 37°C), the culture was refilled to 50 mL with complete RPMI medium, the solution was filtered with a 0.22 µm filter, to remove eggs and larvae that may have remained in the solution, leaving only the antigen. Vivaspin 20 (MWCO 3 kDa, GE, Boston, MA, USA) was used to concentrate *T. canis* antigens, according to manufacturer’s instructions. After centrifugation, the proteic antigen was quantified by the Bradford method and kept at 4°C until use.

For producing bone marrow-derived eosinophils, bone marrow cells were prepared as described above and following the method previously described (22). The cell suspension was resuspended in 3 mL of complete RPMI medium [20% fetal bovine serum (FBS), 2 mM L-glutamine, 1 mM sodium pyruvate, and 100 µL/mL ampicillin] homogenized and transferred to culture bottles. Cells were kept up to day 4 (in 5%CO_2_, 37°C) in RPMI medium supplemented with 100 ng/mL of GM-CSF. Four sets were tested, including both the control group as well as the *T. canis*-infected group with and without antigen stimulation. For bone marrow-derived eosinophil activation, GM-CSF-containing medium was completely replaced with fresh medium containing 10 ng/mL of recombinant murine IL-5 after day 4. Cells were cultured under sterile conditions with supplemented RPMI medium. On the eighth day of culture, cells from the supernatant were centrifuged and transferred to medium bottles and maintained in a fresh medium supplemented with recombinant murine IL-5. Half of the medium was replaced by a new fresh medium every 2 up to 14 days. Upon completion of 14 days of culture, the cells were counted in a Neubauer’s chamber and readjusted to 1 × 10^6^ cells/mL. The expression of various surface and activation molecules was performed by flow cytometric analysis using LSR Fortessa (BD biosciences, San Jose, CA, USA) and using FACS Diva software for acquisition.

### Flow Cytometric Analysis of Eosinophils, B-Cells, Monocyte/Macrophages, and T-Cells

After cell counting and viability assessment, *ex vivo* phenotypic and activation status of APCs and T-cells were evaluated by flow cytometry. For that, one million cells from peripheral blood, spleen, and bone marrow were stained using monoclonal antibodies for phenotypic surface markers: PE anti-Siglec-F (eosinophils), PERCP-Cy5.5 anti-CD19 (B-cells), BV421 anti-F4/80 (monocyte/macrophages) as well as monoclonal antibodies for activation and co-stimulatory-related molecules: FITC anti-CD80, APC anti-CD86, FITC anti-MHC-II, and FITC anti-CD69. T-lymphocytes were evaluated by PE anti-CD3, FITC anti-MHC-II, PE-Cy5 anti-CD28, and FITC anti-CD69. For the *in vitro* studies, one million bone marrow-derived eosinophils were stained for Siglec-F, MHC-II, CD80, CD86, and CD69. For both *ex vivo* and *in vitro* studies, cells were blocked using BALB/c homologous serum (1:20 dilution) for 10 min and washed once prior to staining. All monoclonal antibodies were purchased from BD Pharmingen (San Jose, CA, USA). Antibodies were incubated with cell suspensions for 30 min and washed twice with PBS. Cells were resuspended in PBS and acquired using LSR Fortessa (BD biosciences, San Jose, CA, USA), using FACS Diva Software for acquisition.

### Flow Cytometric Data Analysis

**Figure 1:**
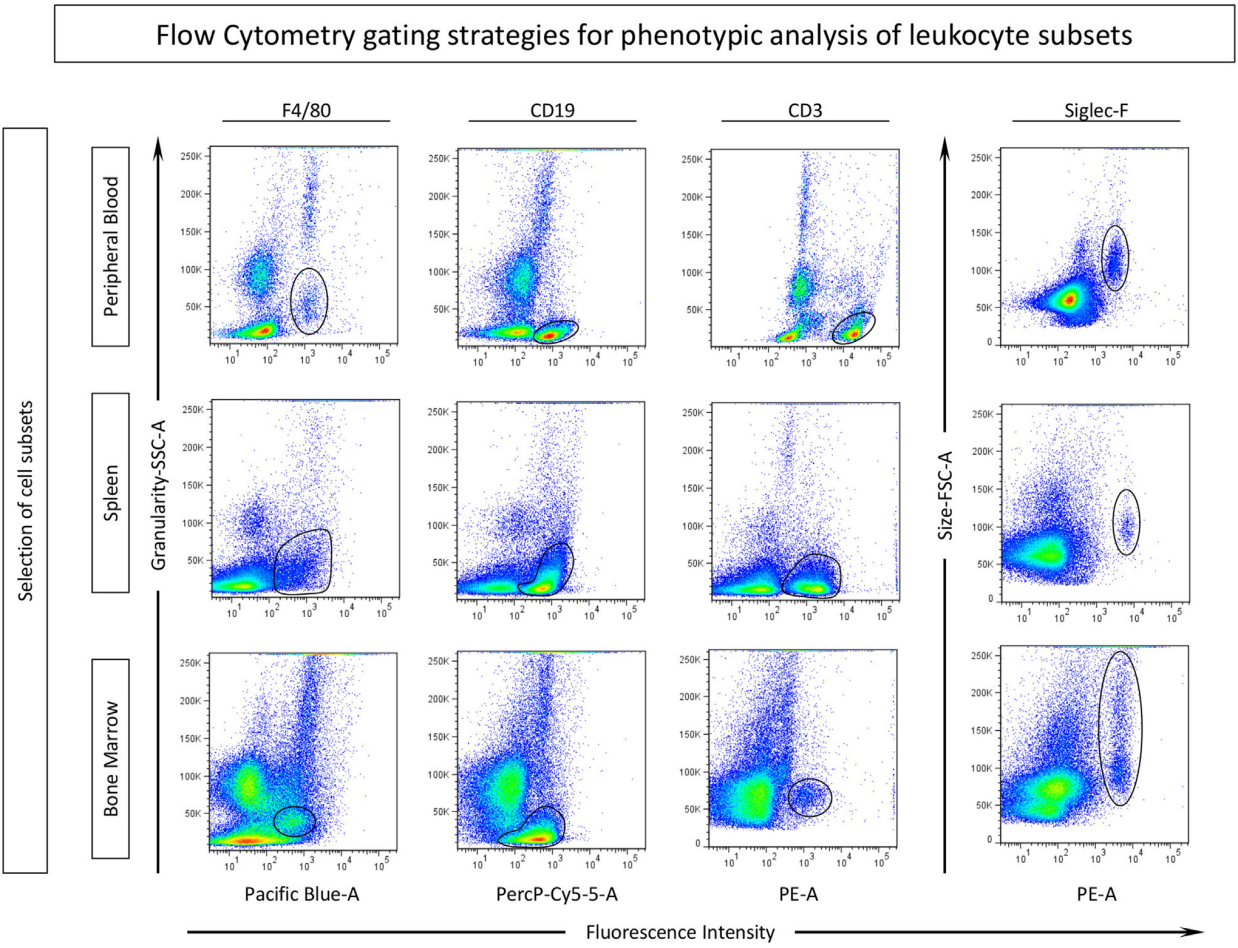

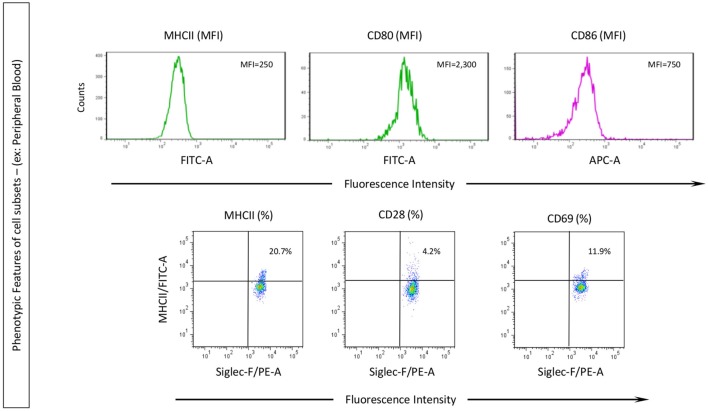
Gating strategies for phenotypic analysis of eosinophils, monocytes, B-cells by flow cytometry. Cell subsets were selected according to their phenotypic expression of surface markers for identifying: monocytes (F4/80), B-cells (CD19), T-cells (CD3), and the target population, eosinophils (Siglec-F), in different compartments: blood, spleen, and bone marrow. Activation and co-stimulatory-related molecules were assessed in each subset by mean fluorescence intensity (MFI) assessed by histograms or percentage of positive cells evaluated by dot plots of these molecules.

Samples were analyzed by the FlowJo software (version 10.0.7, Treestar, San Diego, CA, USA). For *ex vivo* data analysis, gating strategies were performed as illustrated in Figure 1. Cell subsets were defined by fluorescence intensity of F4/80, CD19, and CD3 versus granularity (SSC-A) for assessing the subset of monocytes, B-cells and T-cells, respectively. Eosinophils were selected by Siglec-F versus size (FSC-A). The mean fluorescence intensities (MFI) of CD80, CD86, and MHC-II and the percentage of CD69-positive cells were determined in the selected APC populations (monocytes and B-cells) from peripheral blood, spleen, and bone marrow samples. CD3^+^ T-cells were evaluated by the percentage of CD28 and MHC-II-positive cells. Eosinophils (siglec-F^+^) were evaluated for surface CD80 and CD86 expression (MFI) as well as for the percentage of MHC-II, CD28, and CD69-positive populations (Figure 1).

For *in vitro* flow cytometric data analysis, cells were first gated using pseudocolor plots of size (FSC-H) versus granularity (SSC-H). After that, pseudocolor plots displaying Siglec-F (PE-A) versus size (FSC-H) allowed for selecting two subsets with higher (FSC^HIGH^) and lower (FSC^LOW^) size. Within these two subsets, MHC-II and CD69 were evaluated as percentage and CD80 and CD86 expression was measured by MFI as exemplified in Figure S3 in Supplementary Material.

### Image Flow Cytometry for Confirmation of Activation and Co-Stimulatory Molecule Expression on Eosinophils upon *T. canis* Stimulation

To confirm the presence of bona-fide bone marrow-derived eosinophils and their expression of various surface molecules upon *T. canis* stimulation, cutting-edge image flow cytometric analyses were performed for staining with the monoclonal antibodies: PE anti-Siglec-F^+^, FITC anti-MHC-II, FITC anti-CD69, APC anti-CD86, and FITC anti-CD80 (all purchased from BD Pharmingen). Samples were acquired using ImageStream Mark II and analyzed using Ideas software version 6.1 (both from Millipore-Sigma, Billerica, MA, USA).

### Assessment of Cytokines IL-5, IL-4, and IFN-γ by Immunoenzymatic Assay (ELISA)

ELISA assays to assess IL-5, IL-4, and IFN-γ (BD-Trio Kit IL-5, IL-4, and IFN-γ) (BD Biosciences, San Jose, CA, USA) were carried out according to manufacturer’s instructions. Briefly, 96-well microtiter plates were sensitized with capture antibody pre-diluted in PBS (0.5 mg/mL) using 100 μL/well. Plates were incubated for 18 h at 4°C. The supernatant was discarded, plates were washed with washing solution, and blocked to avoid non-specific binding by adding 200 µL of PBS 10% FBS. Plates were incubated for 1 h at RT and then washed with washing solution. After blocking and washing two times, plates were incubated with pre-diluted plasma samples (1:2) as well as twofold serial dilutions of each standard recombinant cytokine (100 μL/well) starting at 9.76 up to 5,000 pg/mL. After 2-h incubation, a further wash cycle was done and 100 μL/well of the detection antibody (biotinylated secondary antibody at 0.125 mg/mL) was added. After a 1-h incubation at room temperature, a new wash cycle was performed followed by incubation with streptavidin (provided by manufacturer; dilution 1:1,000) diluted in PBS 10% FBS (100 μL/well). After 30 min, the plate was washed again and 100 μL/well of the substrate was added. The substrate used was a 1:1 mixture of H_2_O_2_ and tetramethylbenzidine. Finally, the reaction was stopped by adding 50 μL/well of H_2_SO_4_ (1 M). Absorbance reading was done at 450 nm wavelength in ELISA reader (MicroQuant-Sellex, Inc.).

### Statistical Analysis

The results obtained in the different experiments were normalized through the *Z*-score, interpreted according to the *T* test and expressed as mean ± SEM. For a significance testing, the Mann–Whitney test was used. Statistical significance was considered at *p* < 0.05. The software used was GraphPad Prism 5.0 (San Diego, CA, USA).

### Non-conventional Biomarker Signature Analysis

In this analysis, the whole data universe of every biomarker was used to calculate the global median value, which was used as the cut-off to classify the groups in “*high* expression” or “*low* expression” of a given biomarker. The *high* and *low* expression of every biomarker was selected and the data were used to generate dark- and light-shade diagrams. The diagrams were employed to calculate the frequency of *high* expression in mice for each group. When considering the status of infection, the cut-off point for each biomarker was calculated through the global median of the control group related to the infected group. Then, the relevant data (frequency > 50%) were highlighted in bold. Radar graphs were built to characterize the global frequency of *high* expressing mice in specific biomarkers.

### Network of Correlations between Biomarkers

The Spearman’s correlation test was performed to evaluate the association among the biomarkers tested in non-infected control (NI) and *T. canis*-infected (INF) groups. In all cases, significance was considered at *p* < 0.05. Correlation tests were performed on GraphPad Prism version 5.0 (San Diego, CA, USA). Each correlation was represented by an interaction between two biomarkers using the Cytoscape software (version 2.8; http://www.cytoscape.org), as previously reported (22). The biomarker networks were constructed for NI and INF groups using circular layouts and connections between the biomarkers designated as lines, with thickness according to the correlation index (*r*). The correlation is shown as negative or positive.

## Results

### Phenotypic Profile of Eosinophils, Monocytes, B- and T-Cells upon Experimental *T. canis* Infection in Mice

**Figure 2:**
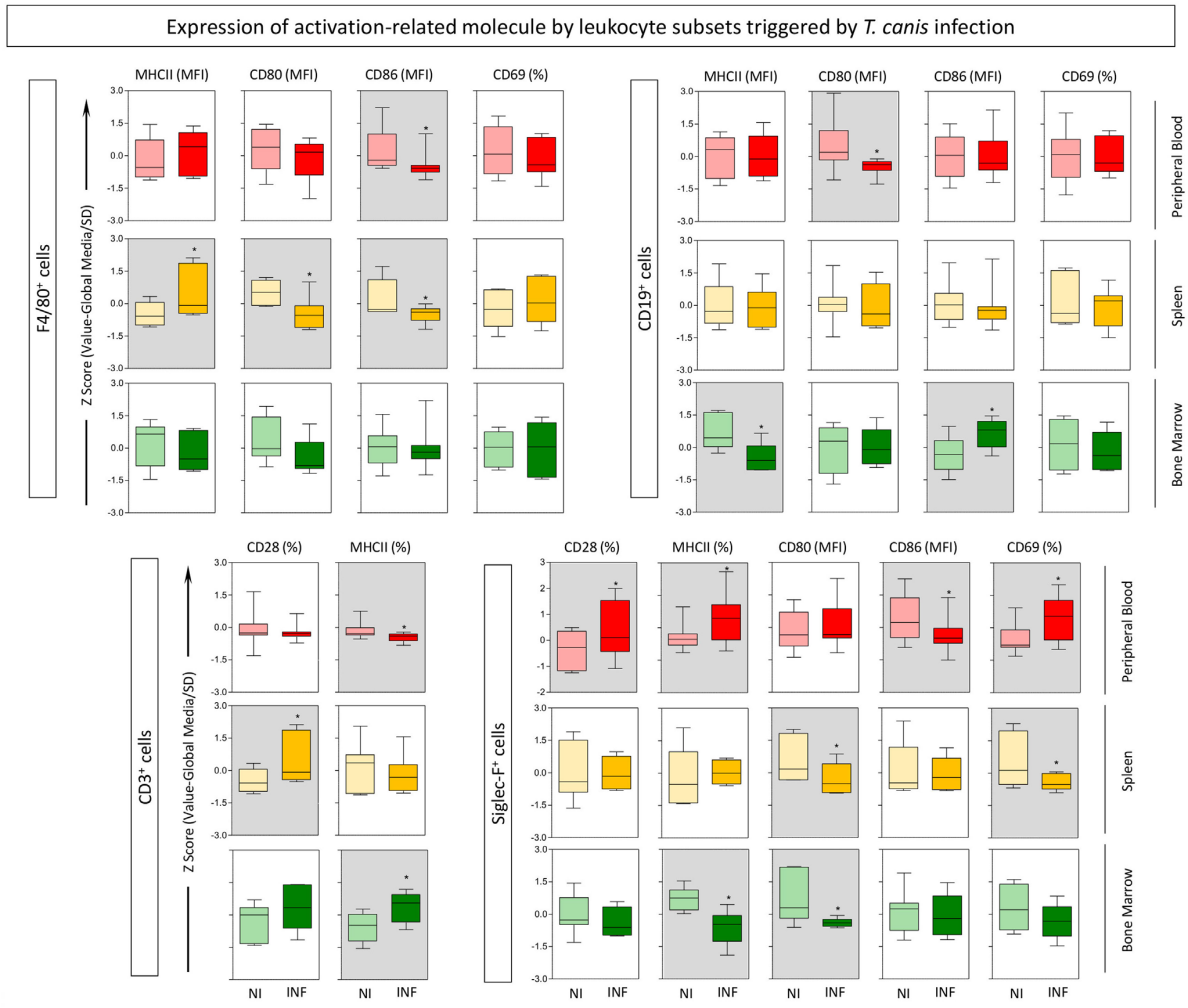
Expression of activation-related molecules by eosinophils, monocytes, B- and T-cells triggered by *Toxocara canis* infection. The global activation profile of F4/80^+^ monocytes, CD19^+^ B-cells, CD3^+^ T-cells, and Siglec-F (eosinophils) was evaluated in *T. canis*-infected (INF) as well as non-infected control (NI) mice. The expression of surface molecules CD28, major histocompatibility complex (MHC-II), CD80, CD86, and CD69 was measured in these subsets from three compartments: blood (red), spleen (yellow), and bone marrow (green). The results are expressed by *Z*-score (value minus global median/SD) of MFI or percentage of positive cells for each cell subset. Statistical differences between NI and INF were highlighted as *p < 0.05.

In order to characterize the role of eosinophils during experimental *T. canis* infection in mice, the phenotypic profile of different leukocyte subsets were evaluated. The gating strategies to define eosinophils, monocytes, B-cells, and T-cells are described in Figure 1. Siglec-F^+^ eosinophils, F4/80^+^ monocytes, CD19^+^ B-cells, and CD3^+^ T-cells were evaluated in three different compartments (peripheral blood, spleen, and bone marrow) in mice infected with *T. canis* as well as NIs. Results are expressed by *Z*-score (value minus global median/SD) of MFI or percentage of positive cells for each cell subset (Figure 2). Data analysis indicated that *T. canis* infection induces significant upregulation of MHC-II and decreased CD80 expression in F4/80^+^ monocytes from spleen. Decreased CD86 expression was observed in this subset from spleen and peripheral blood of *T. canis*-infected mice (Figure 2). In CD19^+^ B-cells, *T. canis* infection promotes a significant decrease in CD80 (peripheral blood) and MHC-II (bone marrow) expression along with an increase in CD86 on B-cells (bone marrow), when comparing with the control group (Figure 2—upper panels). In the CD3^+^ T-cell subset, MHC-II expression was decreased in peripheral blood and increased in the bone marrow. In addition, CD28 expression is increased in splenic CD3^+^ T-cells upon *T. canis* infection as shown in Figure 2 (lower panels).

In the target population, eosinophils (Siglec-F^+^ cells) demonstrated several significant changes upon infection with *T. canis* as follows: CD28, MHC-II, and CD69 expressions are upregulated, while CD86 is downregulated in the peripheral blood; CD80 and CD69 molecules are downregulated in the spleen; MHC-II and CD80 molecules are downregulated in the bone marrow of mice infected with the parasite when compared with the control group.

### Systemic Biomarker Signature Analysis of *T. canis* Infection

In order to understand eosinophil function and their relationship with systemic events occurring in circulating and specific tissues during infection by *T. canis*, a panel containing the proportion of mice with each biomarker expression above the global median cut-off was assembled for peripheral blood, spleen, and bone marrow from both *T. canis*-infected and NI mice.

**Figure 3:**
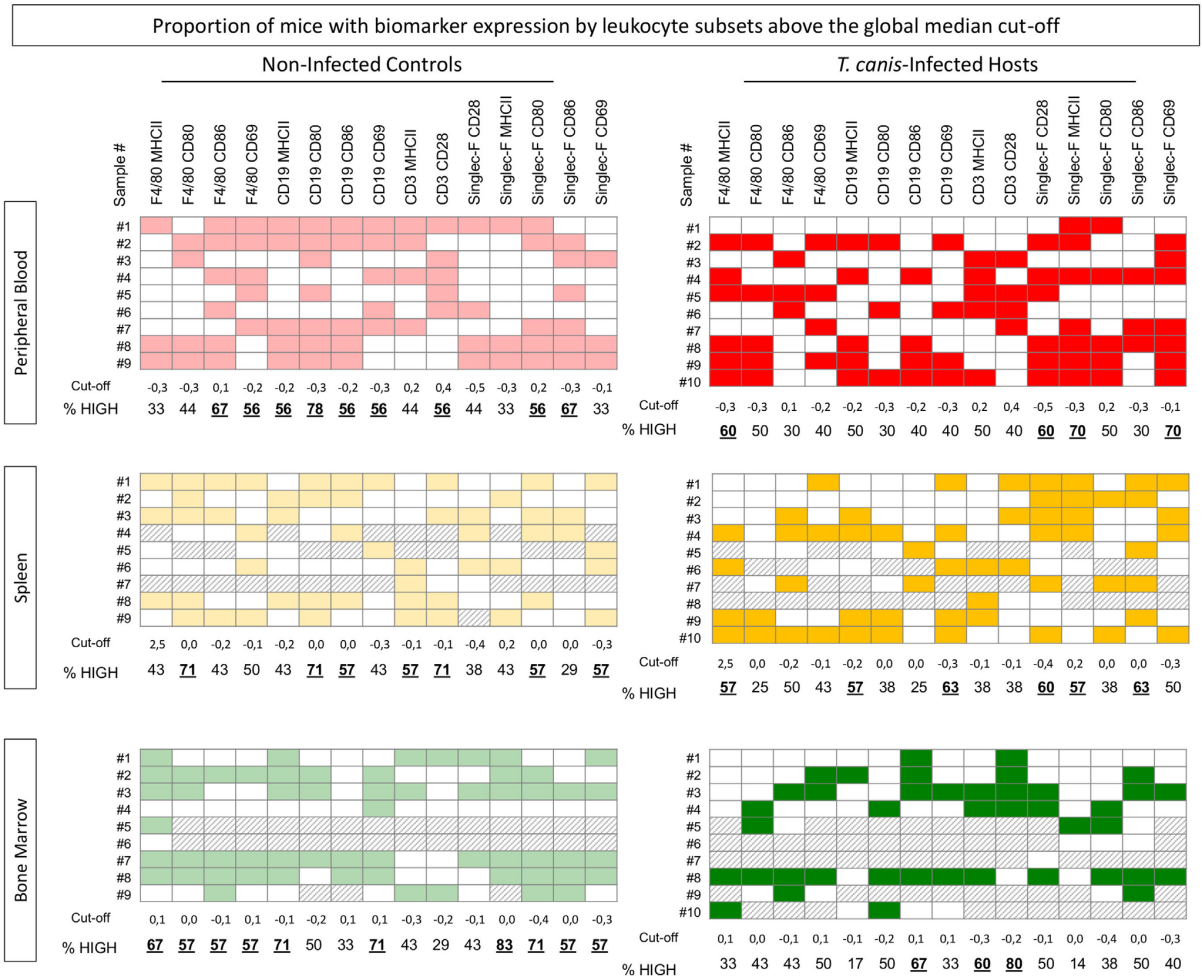
Proportion of mice with biomarker expression by eosinophils, monocytes, B- and T-cells above the global median cut-off. The *Toxocara canis*-infected as well as non-infected control groups were categorized individually as “high-” or “low”-expressing mouse, according to the *Z*-score global median (cut-off) for each activation and co-stimulatory molecule expression on F4/80^+^, CD19^+^, and Siglec-F^+^-cells [major histocompatibility complex (MHC-II), CD80, CD86, and CD69] as well as CD3^+^ T-cells (CD28 and MHC-II) in the three different compartments: blood (red), spleen (yellow), and bone marrow (green). The colored squares in the diagrams represent the “high”-expressing mice, while blank squares represent “low”-expressing mice. The numbers under each column represent the cut-off and the frequency of “high”-expressing mice (%) of each evaluated molecule in each subset.

Figure 3 illustrates the results for the proportion panel analysis, which indicate an overall inversion in the expression profile between NI and INF panels for peripheral blood, spleen, and bone marrow, Interestingly, the proportion of INF with high expression of CD28, MHC-II, and CD69 by peripheral blood eosinophils is increased which is not observed in NI group. On the other hand, the proportion of NI mice with high CD80 and CD86 expression is increased in peripheral blood eosinophils, contrasting with INF mice. A similar result is observed for splenic eosinophils. For bone marrow eosinophils, INF group displayed a decreased proportion of mice with high activation and expression of molecules that was not observed among the NI mice (Figure 3).

**Figure 4:**
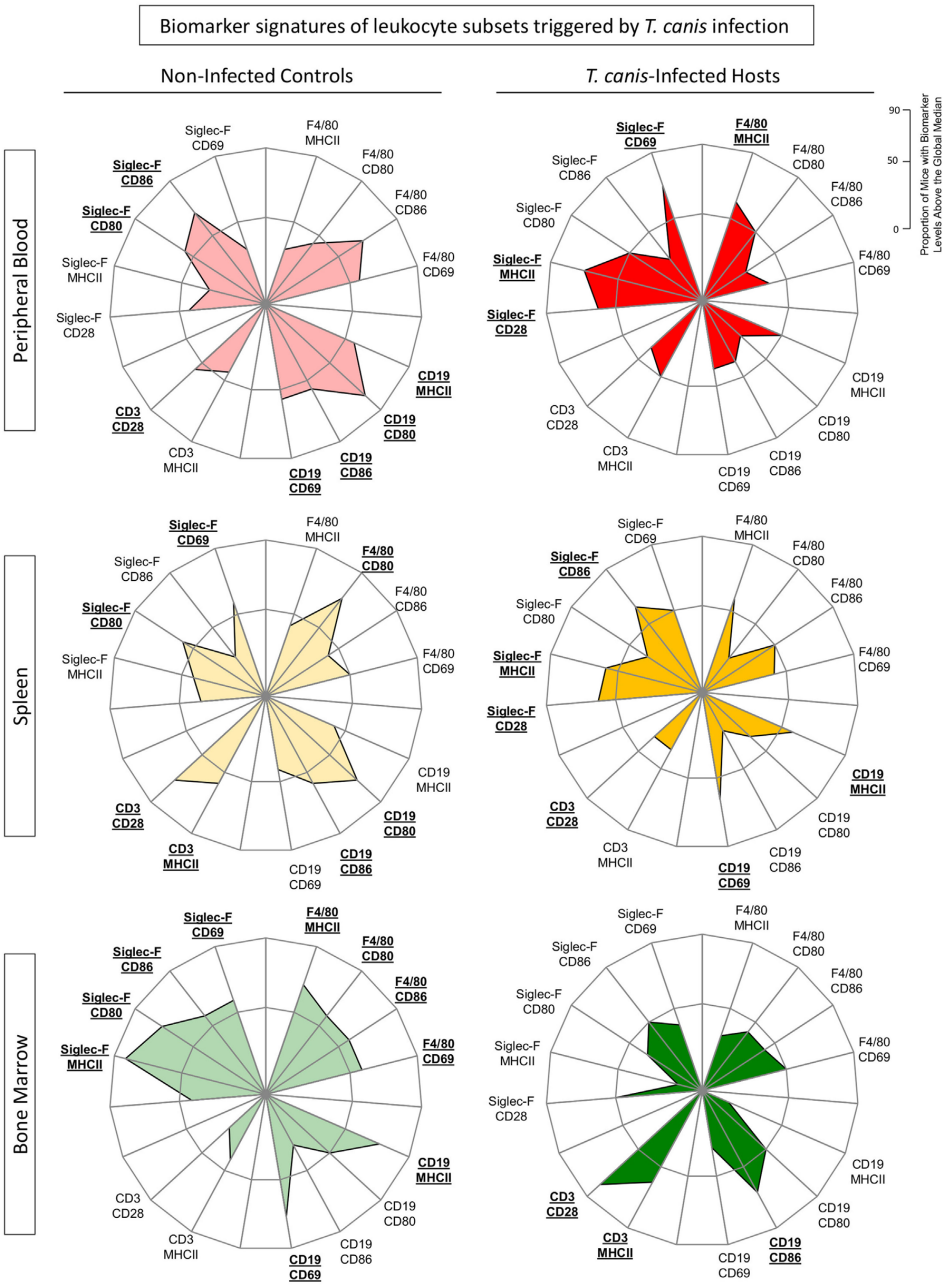
Biomarker signatures of by eosinophils, monocytes, B- and T-cells triggered by *Toxocara canis* infection. The immunological signature of activation and co-stimulatory molecule expression [CD28, CD69, major histocompatibility complex (MHC-II), CD80, and CD86] on F4/80^+^ monocytes, CD19^+^ B-cells, CD3^+^ T-cells, and Siglec-F (eosinophils) was evaluated in *T. canis*-infected as well as non-infected control groups in the three different compartments: blood (red), spleen (yellow), and bone marrow (green). Radar graphs summarize the frequency of “high”-expressing mice (%) in each group. When the frequency of “high”-expressing mice was higher than 50% (on a 0–100% scale), the labels were underlined and highlighted by bold format.

Figure 4 shows the biomarker signatures of eosinophils, monocytes as well as B- and T-cells triggered by *T. canis* infection. A biomarker is credited as important when the frequency of mice with *high* expression of this given parameter was greater than 50% (23). Radar graphs confirm these findings, showing an inversion in the biomarker signature regarding activation and co-stimulatory-related molecule expressions in NI versus INF in all compartments. In peripheral blood and spleen, Siglec-F^+^ eosinophils display an expansion of radar graph area in INF over NI, indicating biomarkers over 50%, especially on MHC-II expression. Conversely, there was a shrinking of radar graph area of INF over NI for all surface molecules tested (CD69, CD80, CD86, and MHC-II) in peripheral blood and splenic B-cells. The opposite effect was observed for Singlec-F^+^ eosinophils and F4/80^+^ cells from bone marrow, with diminished radar graph area, indicating down-modulation of molecule expression specially related to MHC-II in INF when compared with NI.

In spleen, it was also observed a significant increase in Siglec-F^+^CD86^+^ and Siglec-F^+^CD28^+^ as well as CD19 MHC-II^+^/CD69^+^ in the INF compared with NI (Figure 4). In bone marrow, we observed an increased CD3^+^-related graph area in INF when compared with the control group (Figure 4).

### Analysis of Correlations among Immunological Biomarkers: A Systems Biology Approach

**Figure 5:**
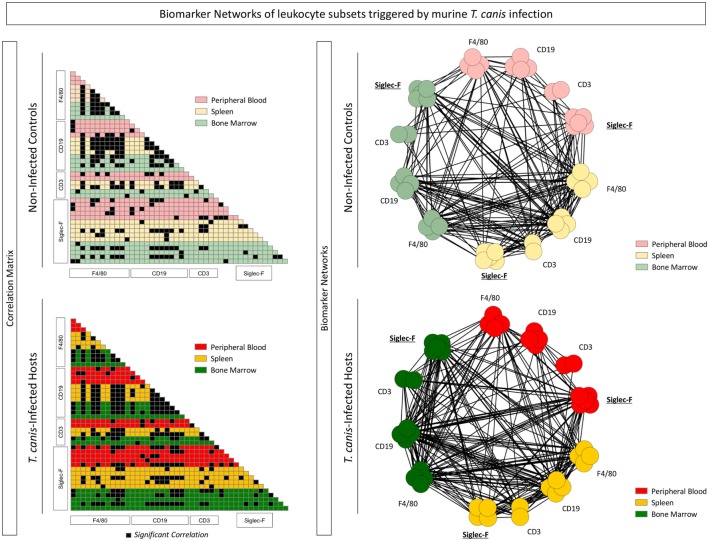
Interaction networks among immunological biomarkers are altered during the *Toxocara canis* infection. Each node in the network represents a biomarker including Siglec-F^+^ eosinophils, F4/80^+^ monocytes, CD19^+^ B-cells, and CD3^+^ T-cells from peripheral blood, spleen, and bone marrow expressing activation and co-stimulatory molecules (CD28, CD69, major histocompatibility complex, CD80, and CD86). Correlations among biomarkers in the different compartments: blood (red), spleen (yellow), and bone marrow (green) were performed and displayed as the color-coded matrices (left panels) and as biomarker networks (right panels). Only significant correlations (*p* < 0.05) for the control (NI) and infected (INF) groups were included in the networks.

To perform a broad multifaceted exploratory analysis and understand the biomarker connectivity upon *T. canis* infection, a correlation matrix was assembled as well as biomarker correlations networks for NI and INF, as shown in Figure 5. Correlations amongst Siglec-F^+^ eosinophils, F4/80^+^ monocytes, CD19^+^ B-cells, and CD3^+^ T-cells from peripheral blood, spleen, and bone marrow were performed and displayed as color-coded matrix (left panels) and as biomarker networks (right panels). Each node in the network represents a biomarker. Positive or negative correlations were subcategorized according to the “strength” of the correlation related to the *r*-value, calculated with Spearman analysis.

Interactions between bone marrow Siglec-F^+^-cell and CD3^+^ T-cell subsets increased in the INF group (Figure 5—left panel). A richly connected network characterized the NI and INF groups. The expression of MHC-II, CD80, CD86, CD69, and CD28 as well as the percentages of Siglec-F^+^-cells, CD19^+^ B-cells, F4/80^+^-cells, and CD3^+^ T-cells correlated strongly to their counterparts among compartments. It was possible to observe that peripheral blood, splenic, and bone marrow Siglec-F^+^ eosinophils from INF group had stronger connections, when compared with the control group (Figure 5—right panel). Using this type of analysis, which considers all data universe, it is possible to highlight the most relevant biomarkers and their relation in the INF group when compared with the control. Thus, the data analysis suggests that Siglec-F^+^ eosinophils could be involved in the immunological events triggered to induce *T. canis*-specific T-cell responses upon infection.

### Impact of *T. canis* Antigen Stimulation *In Vitro* on Cell Surface Molecule Expression by Bone Marrow-Derived Eosinophils

**Figure 6:**
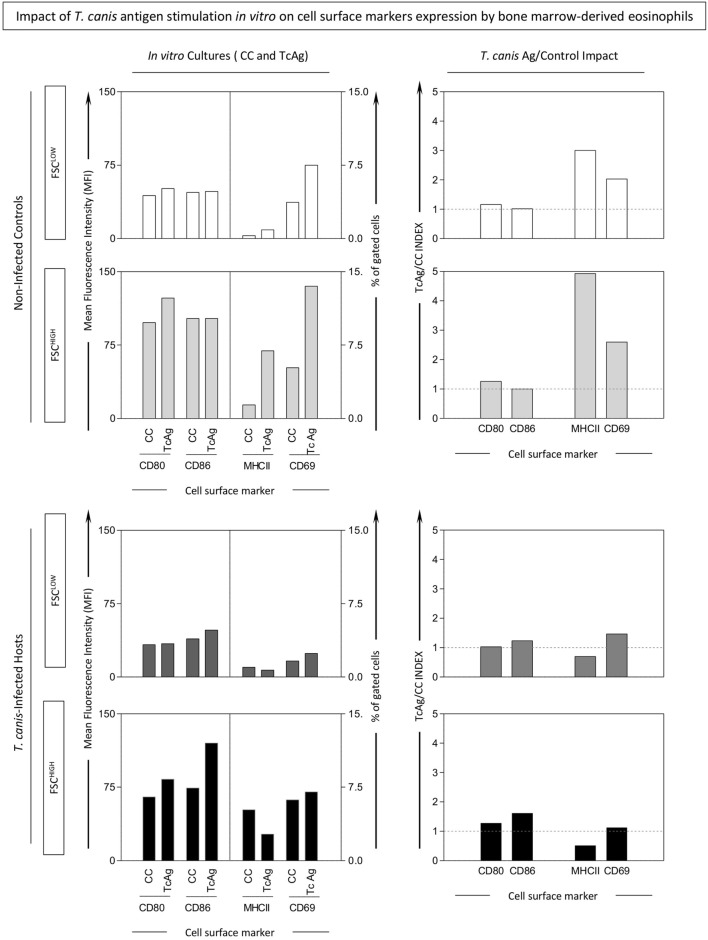
Impact of *in vitro Toxocara canis* antigen stimulation on cell surface molecule expression by bone marrow-derived eosinophils. *In vitro T. canis* antigen stimulation was performed on bone marrow*-*derived eosinophils from *T. canis*-infected as well as non-infected control groups. Control cultures (CC) and antigen-stimulated ones (TcAg) were incubated as described in Section “Materials and Methods” and examined by flow cytometric evaluation of CD69, major histocompatibility complex (MHC-II), CD80, and CD86 within Siglec-F^+^-eosinophils displaying either FSC^HIGH^ (dark shades) or FSC^LOW^ (light shades) phenotype. Activation and antigen presentation-related molecules were also assessed in Siglec-F^+^-eosinophils by mean fluorescence intensity (MFI) or percentage of positive cells and plotted as bar graphs. In addition, fold-changes of TcAg versus CC were calculated to assess the impact of *T. canis* Ag stimuli and expressed as index of stimulation (TcAg/CC INDEX), which was plotted as bar graphs.

Upon differentiation of bone marrow cells from INF and NI into eosinophils, *T. canis* antigen stimulation *in vitro* was performed. After culture, two Siglec-F^+^ subsets were gated for further analysis. These subsets were defined as Siglec-F^+^FSC^HIGH^ and Siglec-F^+^FSC^LOW^, as illustrated in Figure S2 in Supplementary Material. *T. canis* stimulation induced increased percentage of MHC-II^+^Siglec-F^+^FSC^HIGH^ and CD69^+^Siglec-F^+^FSC^HIGH^ in NI mice. No differences were observed in mice infected by *T. canis* (Figure 6).

**Figure 7:**
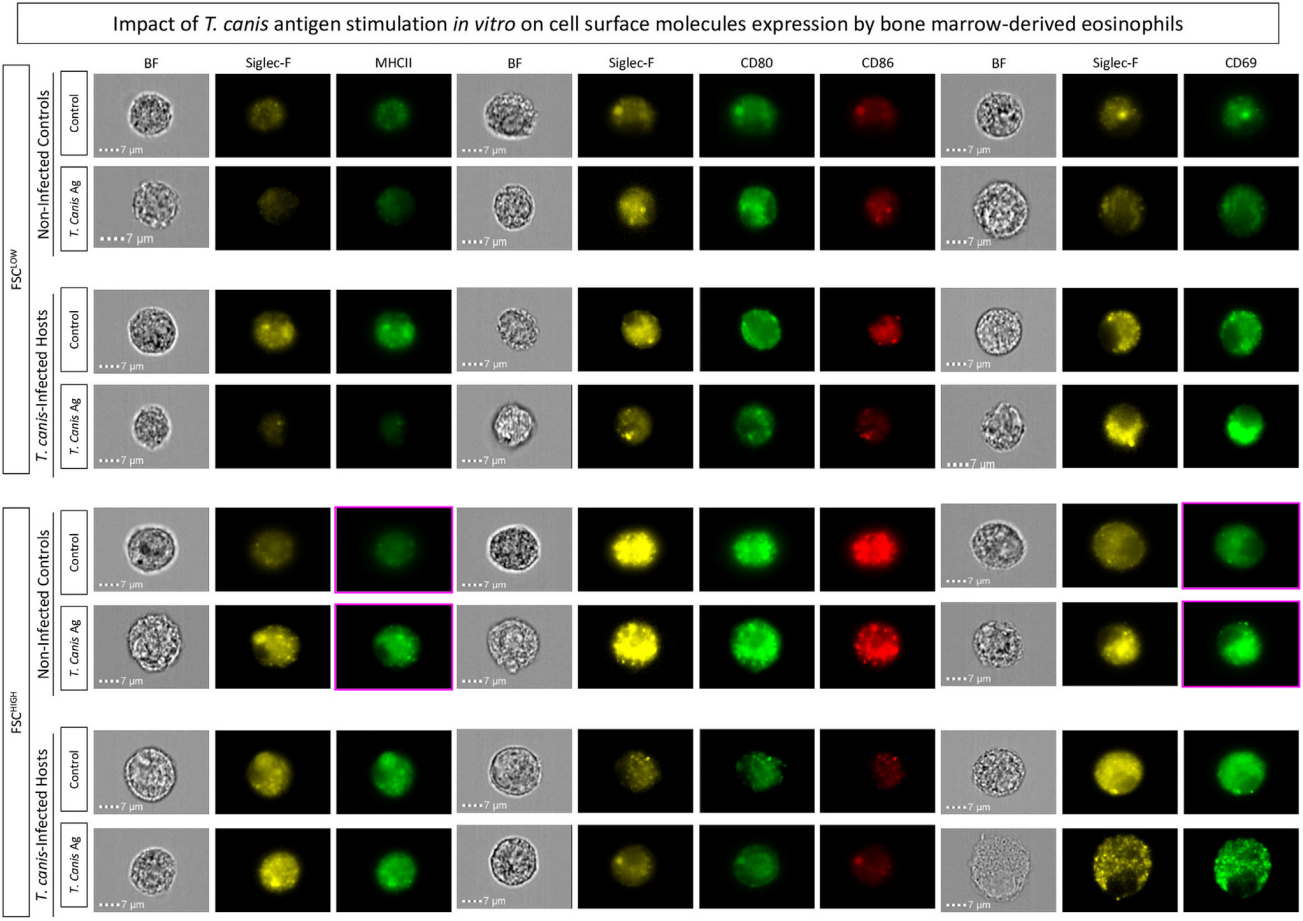
Impact of *in vitro* stimulation with *Toxocara canis* antigens on bone marrow-derived eosinophils. *In vitro T. canis* antigen stimulation was performed on bone marrow*-*derived eosinophils from *T. canis*-infected as well as non-infected control groups. Flow cytometric evaluation of major histocompatibility complex (MHC-II), CD80, CD86, and CD69 within Siglec-F^+^-eosinophils was performed by ImageStream Mark II and analyzed by the Ideas software. Activation and co-stimulatory-related molecules were assessed in Siglec-F^+^-eosinophils by mean fluorescence intensity (MFI) assessed or percentage of positive cells as verified by histograms.

Corroborating these results, image flow cytometric analysis indicated that Siglec-F^+^ eosinophils upregulated MHC-II, and CD69 upon *T. canis* antigen stimuli of bone marrow-derived eosinophils from NIs more evidently than in bone marrow-derived eosinophils from *T. canis*-infected mice (Figure 7).

### Assessing Cytokine Levels in Non-Infected and *T. canis*-Infected Mice Sera by ELISA

After examining the phenotypic profile of Siglec-F^+^ eosinophils upon *T. canis* infection, cytokine production on the 18th day after the *T. canis* infection was evaluated. Eosinophilia peak is observed on the 18th day so it was hypothesized that the circulating cytokines could influence and therefore, be associated with Siglec-F^+^ eosinophil function and activity. Among the main cytokines produced on the eosinophilia peak (18th day), IL-4 showed a significant increase in the INF group when compared with the NI group. IFN-γ and IL-5 production were increased, but significant differences were not observed (Figure S3 in Supplementary Material).

## Discussion

The aim of this study was to determine if the eosinophils could become activated after the exposure to *T. canis* antigens in parallel with antigen-presenting cells (APCs), such as macrophages/monocytes and B-cells. In addition, it was evaluated whether Siglec-F^+^ bone marrow-derived eosinophis would be able to potentially activate upon *T. canis* antigen contact, therefore contributing to the stimulation of immune responses during *T. canis* infections. In order to accomplish these goals, changes in surface markers related to activation were examined in Siglec-F^+^ eosinophis during the experimental *T. canis* infection, along with F4/80^+^-cells, CD19^+^B-cells, and CD3^+^ T-cells. The results demonstrated changes on activation hierarchy during the experimental *T. canis* infection, in which eosinophils are reshaped phenotypically and functionally.

Eosinophils have great importance for the direction of immune response, especially by the cytokine production and as mediators involved directly in the inflammatory process during infections caused by helminthes and allergic diseases (11, 24–26). The activation of immune responses mediated by lymphocytic subsets to specific antigens need the signal resultant from antigen-loaded MHC interaction to its T-cell receptor. In addition, a second signal provided by interactions with co-stimulatory molecules is necessary during the immunological synapse. In addition to MHC-II expression, the capacity to interact and to be able to transmit secondary co-stimulatory activation signals defines professional or conventional APCs (27, 28).

In the toxocariasis context, the assays were performed in order to select populations by gates, especially the target population (eosinophils) and cells that are already considered APC, as monocytes (F4/80) and B-cells (CD19) during the eosinophilia peak on the 18th day.

The data regarding the analyses of the differences in MHC-II expression allows for an evaluation of activation as well as antigen-presentation phenotype. It was possible to observe that Siglec-F^+^ eosinophils had a significant increase in its number in blood and in spleen, when compared with APC cells. These data suggest that the antigenic stimulation of the parasite results in the activation of eosinophils, modifying the role of this cell subset during the murine model of *T. canis* infection.

For a specific activation of the immune response to occur, co-stimulatory molecules in addition to the MHC-II in surface of the APC are necessary (29). Co-stimulatory molecules as CD80 and CD86 (B7.1, B7.2) will interact with CD28 completing the first stage of the antigen presentation, in order to reach a specific immune response. These present data show a significant increase of CD80 in eosinophils in the three compartments (blood, spleen, and bone marrow) of the infected mice when compared with the control group. Similar results were reported in the context of *Strongyloides stercoralis* infection model in which it was observed an expressive increase of the CD80 molecule, along with MHC-II in eosinophils upon infection. The increased expression of the CD80 molecule in eosinophils suggests an efficient activation of the immune response induced by the *T. canis* antigen, considering the potential of CD80 to binds CD28 in T-cell surfaces with high avidity (15, 30).

In regards to activation, increased CD69 expression occurred specifically in the peripheral blood and splenic eosinophils in *T. canis*-infected animals (Figure 2), corroborating with Titz (31) that demonstrated an increase of CD69 expression in peripheral blood eosinophils from patients with atopic dermatitis.

Woerly et al. (32) observed a sensitive increase in CD28 in peripheral blood eosinophils from individuals with hypereosinophilia when compared with healthy individuals, suggesting that these eosinophils may play a role not only in helminthic infections, but also in other disorders associated to allergies, when these cells are generally increased. These data may suggest the participation and activation of eosinophils in determining Th2 polarization during infectious and non-infectious pathologies (33, 34). When analyzing the T-cells population, CD28 expression is increased in spleen and MHC-II upregulated in bone marrow upon *T. canis* infection, indicating productive activation of this subset during peak eosinophilia.

In parallel, F4/80^+^ monocytes were activated conspicuously in a similar manner as eosinophils during infection, which suggests a promiscuous activation induced by *T. canis* infection. In order to make these parameters clearer and more visible, individualized radar and biological signature graphs of each mouse were plotted. The production of IL-5, IL-4, and IFN-γ cytokines in peripheral blood were evaluated on the 18th day after infection, during the peak of eosinophilia (Figure S2 in Supplementary Material). The importance of these data is to reinforce the participation of eosinophils acting significantly in orchestrating Th2 profile responses in a systemic manner. The present data are in agreement with other studies, which demonstrated that, during the *T. canis* infection, the eosinophilia is followed by a significant increase in plasmatic IL-4 levels. These findings suggest that Siglec-F^+^ eosinophils probably require IL-4 not only in the defense against helminthes, but also to exert activation functions (13, 35).

Interestingly, the *in vitro* studies showed that exposure of bone marrow-derived eosinophils to *T. canis* antigens increased significantly the percentage of MHC-II positive as well as CD69^+^ eosinophils in non-infected mice. Other infection models by non-helminth pathogens also support this hypothesis (11, 36, 37). The bone marrow eosinophils from *T. canis*-infected mice did not present any changes in MHC-II expression, even upon *T. canis in vitro* stimulation. This paradoxical effect could be explained by the overactivation of eosinophils during *in vivo* infection that may have induced an anergic state. Studies evaluating *in vitro* eosinophils culture stimulated with tetanus toxin and GM-CSF presented an expressive increase of MHC-II molecule in the stimulated group when compared with the non-stimulated group (8).

Regarding CD69, previous studies corroborate this finding that indicated activation (CD69 upregulation) in eosinophils from mice infected by *S. stercoralis* (38).

At last, the systems biology approaches based on biomarker network and correlation matrices indicated that an enhanced connectivity between eosinophils and T-cells was observed in all three compartments of *T. canis*-infected mice (peripheral blood, spleen, and bone marrow). Moreover, *in vitro T. canis* antigen stimulation resulted in activation and upregulation of activation and co-stimulatory molecules by bone marrow-derived eosinophils. Our findings are evidence of activation and upregulation of important activation and co-stimulatory-related molecules in eosinophils and suggest a reshape of activation hierarchy toward eosinophils during experimental *T. canis* infection.

## Ethics Statement

Avaliação do papel dos eosinófilos como células apresentadoras de antígeno in vitro e ex vivo. Ethics Committee on Animal Experimentation (CEEA) issued by Federal University of São Carlos. approval number 2-058/2013 protocol number 058/2013.

## Author Contributions

JR development of the project, execution of the experiments, and preparation of the manuscript. LC, MA, EF, RC, and DN project execution and data interpretation. JR content and language review. OM-F, AT-C, and FA substantial contributions to the conception or design of the work; the acquisition, analysis, and interpretation of data for the work.

## Conflict of Interest Statement

The authors declare that the research was conducted in the absence of any commercial or financial relationships that could be construed as a potential conflict of interest.
