# Supplementary material for: Robust Phenotypic Activation of Eosinophils during Experimental Toxocara canis Infection
Source: Front Immunol. 2018 Jan 31;9:64. doi: 10.3389/fimmu.2018.00064 (PMC5797789; doi:10.3389/fimmu.2018.00064)
Supplement: Supplementary file 1 [file Data_Sheet_1.docx]

Supplementary Material

**Robust Phenotypic Activation of Eosinophils during Experimental *Toxocara canis* infection**

Joice Margareth de Almeida Rodolpho*^1^, Luciana Camillo^1^, Marcio Sobreira Silva Araujo^2^, Elaine Speziali de Faria^2^, Jordana Grazziela Coelho-dos-Reis^2^, Ricardo de Oliveira Correia^1^, Débora Meira Neris^1^, Olindo Assis Martins-Filho^2^, Andréa Teixeira-Carvalho^2^, Fernanda de Freitas Anibal^1^

^1^ Laboratory of Inflammation and Infectious Diseases, Department of Morphology and Pathology, Federal University of São Carlos-UFSCar- São Carlos (SP), Brazil.

^2^Integrated Research Group on Biomarkers of Research René Rachou-Fiocruz- Belo Horizonte (MG), Brazil.

Correspondece:

*Joice Margareth de Almeida rodolpho [J_Jrodolpho@hotmail.com](mailto:J_Jrodolpho@hotmail.com)

**Supplementary Figure 1: Experimental design.** The strategy used for the experimental *T. canis* infection as well as *ex vivo* and *in vitro* studies were organized in a flowchart.

**Supplementary Figure 2: Flow cytometric results for bone marrow*-*derived eosinophil activation profile upon *T. canis* antigen-induced recall *in vitro*.** *In vitro* *T. canis* antigen stimulation was performed on Bone marrow*-*derived eosinophils from *T. canis*-infected (INF) as well as non-infected control (NI) groups. Flow cytometric evaluation of CD69, MHC-II, CD80 and CD86 within Siglec-F+-eosinophils displaying either FSC^HIGH^ or FSC^LOW^ phenotype. Activation and co-stimulatory-related molecules were assessed in Siglec-F^+^-eosinophils by Mean Fluorescence Intensity (MFI) or percentage of positive cells as verified by histograms.

**Supplementary Figure 3: Cytokine levels in serum samples of *Toxocara canis-infected and non-infected mice*.** The levels of IFN-γ, IL-4 and IL-5 were evaluated in the serum of mice from control group (white) and infected group (black). The results are expressed in Median. Statistical significance are indicated as * p<0.05.
