# Supplementary figures and images for: Robust Phenotypic Activation of Eosinophils during Experimental Toxocara canis Infection
Source: Front Immunol. 2018 Jan 31;9:64. doi: 10.3389/fimmu.2018.00064 (PMC5797789; doi:10.3389/fimmu.2018.00064)

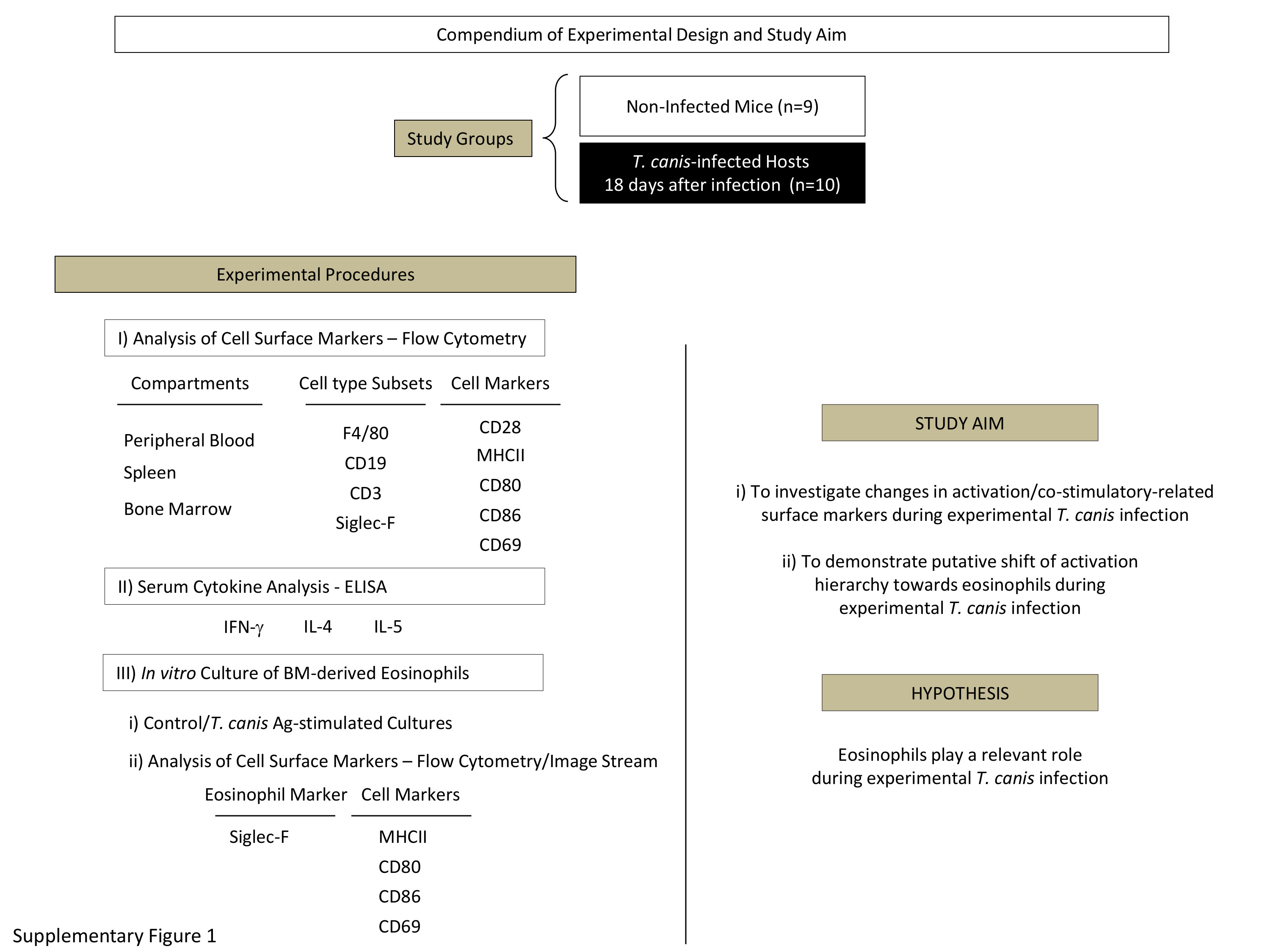

Supplement: Supplementary file 2 [file Image_1.jpeg]

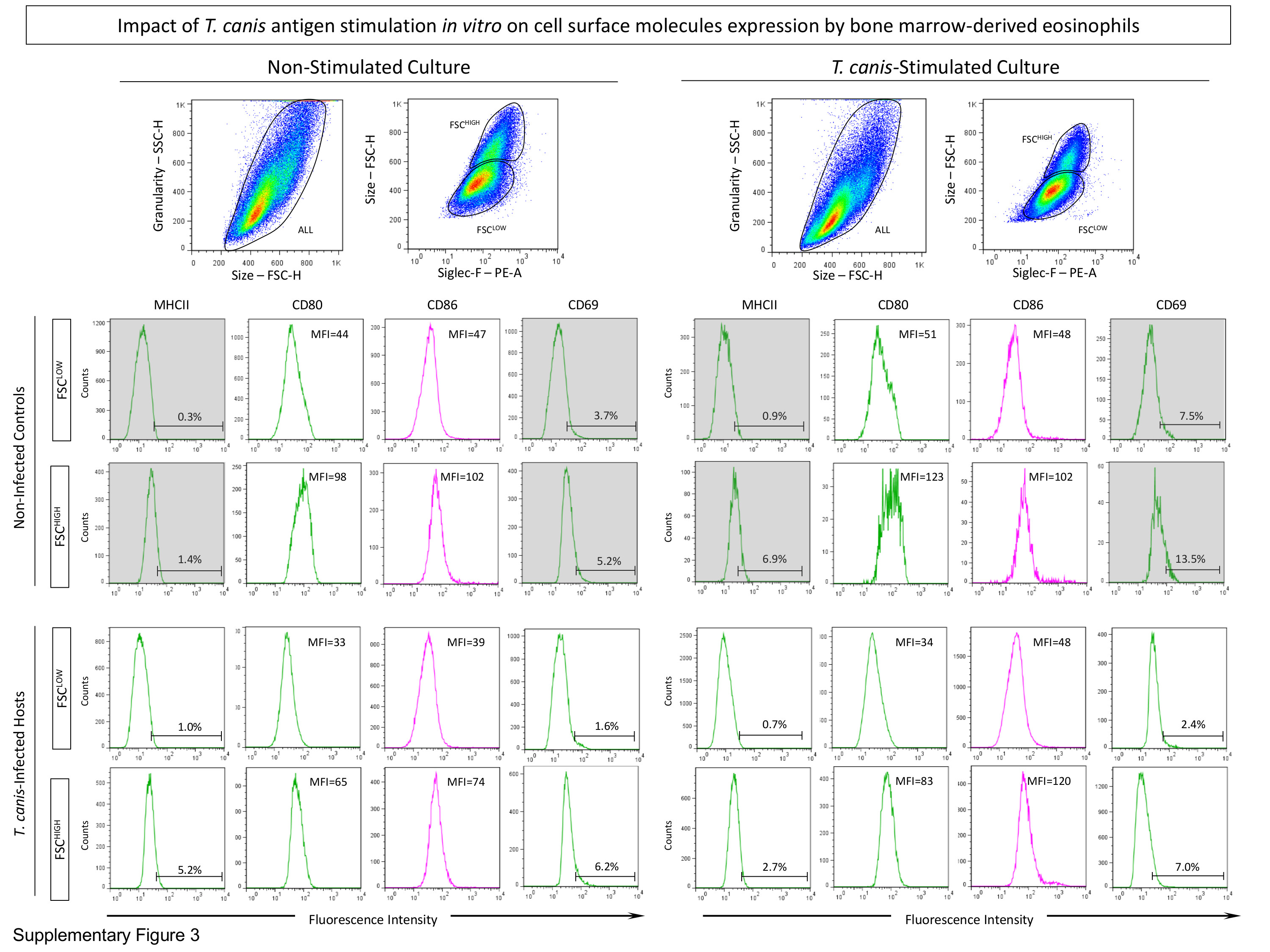

Supplement: Supplementary file 3 [file Image_2.jpeg]

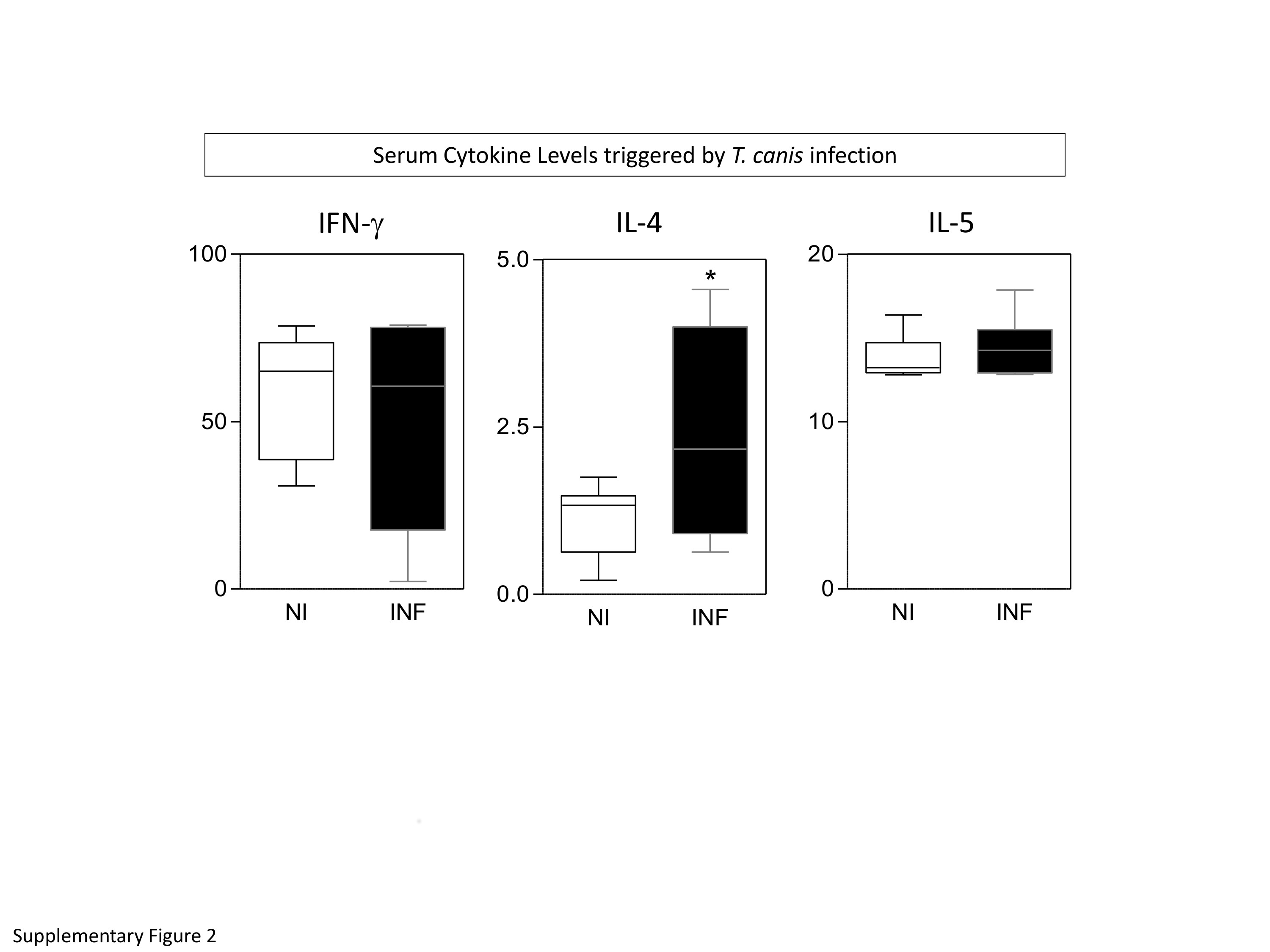

Supplement: Supplementary file 4 [file Image_3.jpeg]
